# 1,1,4,7,7-Penta­methyl­diethylenetri­ammonium trinitrate

**DOI:** 10.1107/S1600536814001469

**Published:** 2014-01-25

**Authors:** Sofian Gatfaoui, Mohamed Rzaigui, Houda Marouani

**Affiliations:** a Laboratoire de Chimie des Matériaux, Faculté des Sciences de Bizerte, 7021 Zarzouna Bizerte, Tunisia

## Abstract

In the title compound, C_9_H_26_N_3_^3+^·3NO_3_^−^, the triprotonated 1,1,4,7,7-penta­methyl­diethylenetri­amine mol­ecules are linked to the nitrate anions by multiple bifurcated N—H⋯(O,O) and weak C—H⋯O hydrogen bonds. The organic cation is characterized by N—C—C—N torsion angles of −176.2 (2) and 176.6 (2)°.

## Related literature   

For related structures, see: Marouani *et al.* (2012 ▶); Gatfaoui *et al.* (2013 ▶); Kefi *et al.* (2013 ▶); Ben Slimane & Smirani (2008 ▶); Morawitz *et al.* (2005 ▶). For a discussion on hydrogen bonding, see: Brown (1976 ▶); Blessing (1986 ▶).
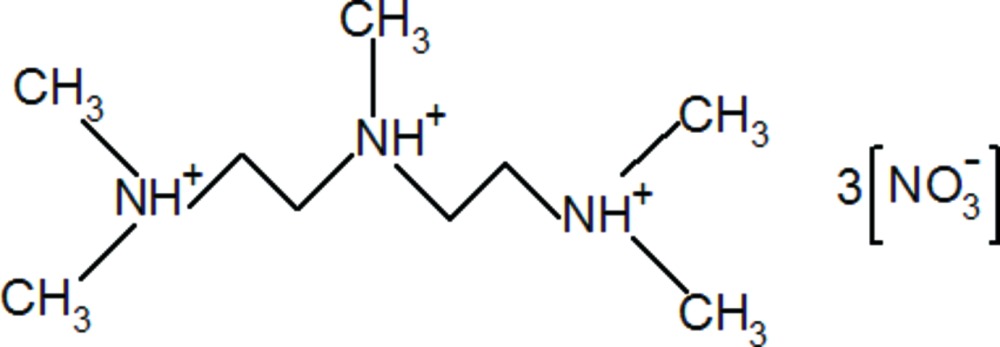


## Experimental   

### 

#### Crystal data  

C_9_H_26_N_3_^3+^·3NO_3_^−^
*M* *_r_* = 362.36
Triclinic, 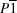

*a* = 5.964 (2) Å
*b* = 7.018 (1) Å
*c* = 21.688 (2) Å
α = 91.90 (2)°
β = 90.60 (2)°
γ = 102.45 (3)°
*V* = 885.8 (3) Å^3^
*Z* = 2
Ag *K*α radiation
λ = 0.56083 Å
μ = 0.07 mm^−1^
*T* = 293 K
0.40 × 0.35 × 0.30 mm

#### Data collection  

Enraf–Nonius CAD4 diffractometer
3582 measured reflections
3128 independent reflections
2125 reflections with *I* > 2σ(*I*)
*R*_int_ = 0.030
2 standard reflections every 120 min intensity decay: 2%

#### Refinement  

*R*[*F* ^2^ > 2σ(*F* ^2^)] = 0.063
*wR*(*F* ^2^) = 0.184
*S* = 1.05
3128 reflections
234 parameters
H atoms treated by a mixture of independent and constrained refinement
Δρ_max_ = 0.29 e Å^−3^
Δρ_min_ = −0.24 e Å^−3^

### 

Data collection: *CAD-4 EXPRESS* (Enraf–Nonius, 1994 ▶); cell refinement: *CAD-4 EXPRESS*; data reduction: *XCAD4* (Harms & Wocadlo, 1995 ▶); program(s) used to solve structure: *SHELXS97* (Sheldrick, 2008 ▶); program(s) used to refine structure: *SHELXL97* (Sheldrick, 2008 ▶); molecular graphics: *ORTEP-3 for Windows* (Farrugia, 2012 ▶) and *DIAMOND* (Brandenburg & Putz, 2005 ▶); software used to prepare material for publication: *WinGX* publication routines (Farrugia, 2012 ▶).

## Supplementary Material

Crystal structure: contains datablock(s) I, global. DOI: 10.1107/S1600536814001469/bh2492sup1.cif

Structure factors: contains datablock(s) I. DOI: 10.1107/S1600536814001469/bh2492Isup2.hkl

Supporting information file. DOI: 10.1107/S1600536814001469/bh2492Isup3.cml

CCDC reference:

Additional supporting information:  crystallographic information; 3D view; checkCIF report

## Figures and Tables

**Table 1:** Hydrogen-bond geometry (Å, °)

*D*—H⋯*A*	*D*—H	H⋯*A*	*D*⋯*A*	*D*—H⋯*A*
N1—H1⋯O9^i^	0.90 (3)	2.04 (3)	2.868 (4)	153 (3)
N1—H1⋯O7^i^	0.90 (3)	2.14 (3)	2.937 (4)	147 (3)
N2—H2⋯O5	0.87 (3)	1.90 (3)	2.749 (3)	162 (3)
N2—H2⋯O4	0.87 (3)	2.42 (3)	3.134 (4)	139 (2)
N3—H3⋯O1^i^	0.87 (3)	2.00 (3)	2.789 (4)	149 (3)
N3—H3⋯O3^i^	0.87 (3)	2.28 (3)	3.073 (4)	152 (3)
C1—H1*A*⋯O7^ii^	0.97	2.38	3.293 (4)	156
C1—H1*B*⋯O4	0.97	2.45	3.232 (4)	137
C2—H2*B*⋯O5^iii^	0.97	2.52	3.386 (4)	149
C3—H3*B*⋯O1^ii^	0.97	2.44	3.308 (4)	148
C4—H4*A*⋯O5^iii^	0.97	2.40	3.301 (4)	155
C5—H5*C*⋯O8^iv^	0.96	2.40	3.305 (5)	156
C7—H7*A*⋯O6^v^	0.96	2.42	3.295 (4)	152
C8—H8*A*⋯O2^iv^	0.96	2.50	3.355 (5)	149
C9—H9*A*⋯O2^iv^	0.96	2.53	3.377 (5)	148

Symmetry codes: (i) 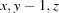; (ii) 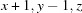; (iii) 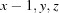; (iv) 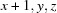; (v) 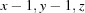.

**Fig. 1.**
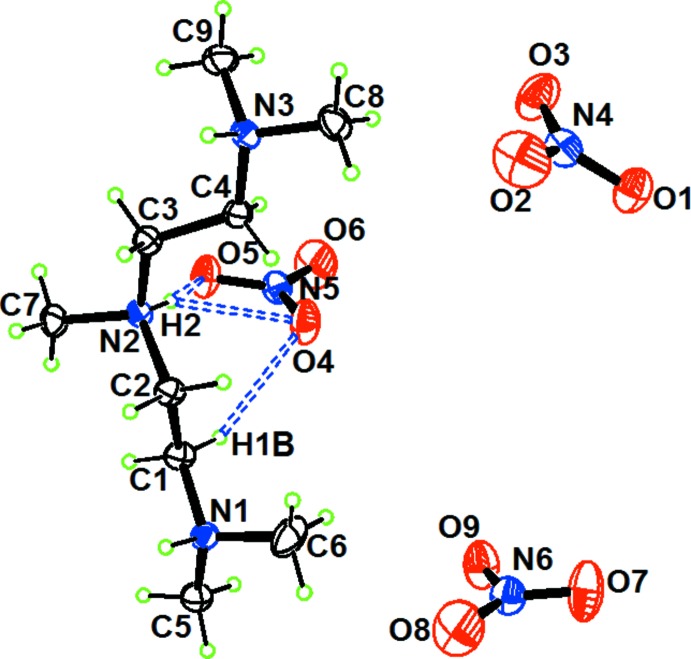
An ORTEP view of the asymmetric unit of the title compound, with displacement ellipsoids drawn at the 30% probability level. H atoms are represented as small spheres of arbitrary radii. Hydrogen bonds are shown as dotted lines.

**Fig. 2.**
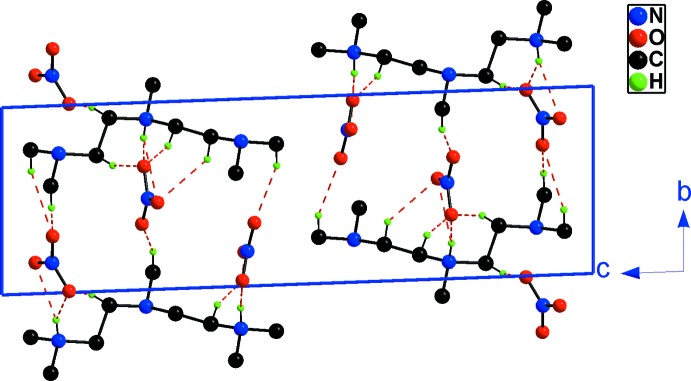
Projection of the title compound along the a axis. The H atoms not involved in H-bonding are omitted.

## supplementary crystallographic information

### 1. Comment

1,1,4,7,7-Pentamethyldiethylenetriamine is an amine catalyst primarily used in the production of spray polyurethane foam (SPF). It is classified as tertiary and has basic and nucleophilic properties. In this work, we report the preparation and the structural investigation of a new organic nitrate, C_9_H_26_N_3_(NO_3_)_3_.

The asymmetric unit of the title salt consists of three nitrate anions and one 1,1,4,7,7-pentamethylethylenetriammonium trication (Fig. 1). The organic cations are connected to the nitrate anions through multiple bifurcated N—H···O and weak C—H···O hydrogen bonds, with donor-acceptor distances varying between 2.749 (3) and 3.386 (4) Å [d (O···O) > 2.73 Å; Brown, 1976; Blessing, 1986. See Table 1 and Fig. 2).

Nitrate anions are distributed in the (*b*,*c*) plane and are interconnected through the organic cations which extend in the direction of the *c* axis. Geometrical characteristics of the three independent nitrate anions are slightly different. The distance N5—O6 is notably the shortest, 1.204 (3) Å, because O6 is applied in only one hydrogen bond (Table 1) at the same time as N5—O5 distance is much larger, 1.248 (3) Å, because O5 is involved in three hydrogen bonds. These geometrical features have also been noticed in other crystal structures (Marouani *et al.*, 2012; Gatfaoui *et al.*, 2013; Kefi *et al.*, 2013).

Each organic entity is bounded to six different nitrate anions through fifteen N—H···O and C—H···O hydrogen bonds forming a two dimensional network. Examination of the 1,1,4,7,7-pentamethylethylenetriammonium cation shows that the bond distances and angles show no significant difference from those obtained in other structures involving the same organic groups (Morawitz *et al.*, 2005; Ben Slimane & Smirani, 2008). The crystal cohesion and stability are ensured by electrostatic and van der Waals interactions which, together with N—H···O and C—H···O hydrogen bonds, build up a two-dimensional network.

### 2. Experimental

An aqueous solution containing 3 mmol of HNO_3_ in 10 ml of water was added to 1 mmol of 1,1,4,7,7-pentamethylethylenetriamine in 10 ml of water. The resulting solution was stirred for 15 min. and then left to stand at room temperature. Colourless single crystals of the title compound were obtained after some days.

### 3. Refinement

The hydrogen atoms bonded to N1, N2 and N3 were located in a difference map and were allowed to refine (coordinates and isotropic displacement parameters). The rest of the H atoms were treated as riding, with C—H = 0.97 Å (methylene) or 0.96 Å (methyl), and with *U*_iso_(H) = 1.2*U*_eq_(C) or *U*_iso_(H) = 1.5*U*_eq_(C).

### Crystal data

**Table:**

C_9_H_26_N_6_O_9_	*Z* = 2
*M**_r_* = 362.36	*F*(000) = 388
Triclinic, *P*1	*D*_x_ = 1.359 Mg m^−^^3^
Hall symbol: -P 1	Ag *K*α radiation, λ = 0.56083 Å
*a* = 5.964 (2) Å	Cell parameters from 25 reflections
*b* = 7.018 (1) Å	θ = 9–11°
*c* = 21.688 (2) Å	µ = 0.07 mm^−^^1^
α = 91.90 (2)°	*T* = 293 K
β = 90.60 (2)°	Prism, colourless
γ = 102.45 (3)°	0.40 × 0.35 × 0.30 mm
*V* = 885.8 (3) Å^3^	

### Data collection

**Table:**

Enraf–Nonius CAD4 diffractometer	*R*_int_ = 0.030
Radiation source: fine-focus sealed tube	θ_max_ = 19.5°, θ_min_ = 2.2°
Graphite monochromator	*h* = −7→7
non–profiled ω scans	*k* = −8→8
3582 measured reflections	*l* = −2→25
3128 independent reflections	2 standard reflections every 120 min
2125 reflections with *I* > 2σ(*I*)	intensity decay: 2%

### Refinement

**Table:**

Refinement on *F*^2^	Primary atom site location: structure-invariant direct methods
Least-squares matrix: full	Secondary atom site location: difference Fourier map
*R*[*F*^2^ > 2σ(*F*^2^)] = 0.063	Hydrogen site location: inferred from neighbouring sites
*wR*(*F*^2^) = 0.184	H atoms treated by a mixture of independent and constrained refinement
*S* = 1.05	*w* = 1/[σ^2^(*F*_o_^2^) + (0.0789*P*)^2^ + 0.6513*P*] where *P* = (*F*_o_^2^ + 2*F*_c_^2^)/3
3128 reflections	(Δ/σ)_max_ < 0.001
234 parameters	Δρ_max_ = 0.29 e Å^−^^3^
0 restraints	Δρ_min_ = −0.24 e Å^−^^3^
0 constraints	

### Fractional atomic coordinates and isotropic or equivalent isotropic displacement parameters (Å^2^)

**Table:**

	*x*	*y*	*z*	*U*_iso_*/*U*_eq_	
N1	0.4083 (4)	0.2269 (4)	0.40433 (11)	0.0411 (6)	
N2	0.5838 (4)	0.0897 (3)	0.24407 (10)	0.0344 (5)	
N3	0.3516 (4)	0.2565 (4)	0.09611 (11)	0.0388 (6)	
N4	−0.0530 (5)	0.8384 (4)	0.08493 (13)	0.0548 (7)	
N5	0.9855 (5)	0.5138 (4)	0.24709 (13)	0.0519 (7)	
N6	0.0036 (5)	0.8141 (4)	0.41497 (14)	0.0601 (7)	
O1	−0.0634 (4)	0.9934 (4)	0.11371 (13)	0.0711 (7)	
O2	−0.2111 (5)	0.6968 (5)	0.08600 (17)	0.1129 (12)	
O3	0.1225 (5)	0.8341 (4)	0.05677 (15)	0.0926 (10)	
O4	0.7958 (5)	0.5372 (4)	0.26319 (15)	0.0839 (9)	
O5	1.0059 (4)	0.3412 (3)	0.23999 (13)	0.0675 (7)	
O6	1.1418 (5)	0.6488 (5)	0.23730 (18)	0.1128 (12)	
O7	−0.0504 (5)	0.9697 (5)	0.40675 (18)	0.1064 (11)	
O8	−0.1275 (6)	0.6643 (5)	0.4269 (2)	0.1306 (15)	
O9	0.2109 (4)	0.8175 (4)	0.41010 (15)	0.0816 (8)	
C1	0.5618 (5)	0.1954 (4)	0.35333 (13)	0.0433 (7)	
H1A	0.6433	0.0955	0.3644	0.052*	
H1B	0.6746	0.3152	0.3474	0.052*	
C2	0.4284 (4)	0.1337 (4)	0.29386 (13)	0.0411 (7)	
H2A	0.3090	0.0186	0.3003	0.049*	
H2B	0.3553	0.2373	0.2812	0.049*	
C3	0.4709 (5)	0.0753 (4)	0.18173 (13)	0.0395 (7)	
H3A	0.3187	−0.0081	0.1834	0.047*	
H3B	0.5593	0.0157	0.1524	0.047*	
C4	0.4516 (5)	0.2739 (4)	0.15978 (13)	0.0424 (7)	
H4A	0.3550	0.3304	0.1875	0.051*	
H4B	0.6026	0.3600	0.1603	0.051*	
C5	0.5362 (6)	0.2467 (5)	0.46408 (14)	0.0561 (8)	
H5A	0.6040	0.1357	0.4688	0.084*	
H5B	0.4326	0.2537	0.4972	0.084*	
H5C	0.6548	0.3636	0.4649	0.084*	
C6	0.3009 (7)	0.3951 (6)	0.39551 (19)	0.0763 (12)	
H6A	0.4184	0.5104	0.3903	0.114*	
H6B	0.2120	0.4134	0.4310	0.114*	
H6C	0.2027	0.3708	0.3595	0.114*	
C7	0.6704 (5)	−0.0917 (4)	0.25532 (15)	0.0485 (8)	
H7A	0.5432	−0.2019	0.2553	0.073*	
H7B	0.7494	−0.0783	0.2945	0.073*	
H7C	0.7740	−0.1109	0.2233	0.073*	
C8	0.2505 (6)	0.4277 (5)	0.08285 (16)	0.0559 (8)	
H8A	0.3696	0.5443	0.0843	0.084*	
H8B	0.1376	0.4393	0.1131	0.084*	
H8C	0.1794	0.4099	0.0426	0.084*	
C9	0.5213 (6)	0.2317 (5)	0.04887 (15)	0.0597 (9)	
H9A	0.6439	0.3457	0.0496	0.090*	
H9B	0.4479	0.2142	0.0089	0.090*	
H9C	0.5821	0.1193	0.0575	0.090*	
H1	0.304 (6)	0.114 (5)	0.4038 (14)	0.051 (9)*	
H2	0.701 (5)	0.188 (4)	0.2414 (12)	0.036 (8)*	
H3	0.247 (6)	0.148 (5)	0.0925 (14)	0.051 (9)*	

### Atomic displacement parameters (Å^2^)

**Table:**

	*U*^11^	*U*^22^	*U*^33^	*U*^12^	*U*^13^	*U*^23^
N1	0.0408 (13)	0.0376 (13)	0.0435 (14)	0.0050 (11)	0.0083 (11)	−0.0007 (10)
N2	0.0270 (11)	0.0358 (13)	0.0420 (13)	0.0093 (10)	0.0055 (10)	0.0069 (10)
N3	0.0332 (12)	0.0402 (14)	0.0420 (14)	0.0044 (10)	0.0009 (10)	0.0080 (10)
N4	0.0470 (16)	0.0527 (17)	0.0597 (17)	−0.0003 (13)	−0.0091 (14)	0.0049 (13)
N5	0.0464 (16)	0.0452 (16)	0.0606 (17)	0.0017 (13)	−0.0009 (13)	0.0042 (12)
N6	0.0506 (17)	0.0542 (18)	0.073 (2)	0.0052 (14)	0.0031 (14)	0.0033 (14)
O1	0.0511 (14)	0.0646 (16)	0.097 (2)	0.0111 (12)	0.0169 (13)	−0.0059 (14)
O2	0.088 (2)	0.092 (2)	0.129 (3)	−0.0453 (18)	−0.0175 (19)	0.0045 (19)
O3	0.083 (2)	0.087 (2)	0.110 (2)	0.0251 (16)	0.0385 (18)	−0.0092 (17)
O4	0.0724 (18)	0.0576 (16)	0.131 (2)	0.0310 (13)	0.0247 (17)	0.0134 (15)
O5	0.0396 (12)	0.0509 (14)	0.114 (2)	0.0139 (10)	0.0117 (12)	0.0000 (13)
O6	0.084 (2)	0.074 (2)	0.158 (3)	−0.0324 (17)	0.019 (2)	0.003 (2)
O7	0.083 (2)	0.082 (2)	0.166 (3)	0.0474 (17)	−0.029 (2)	−0.004 (2)
O8	0.099 (2)	0.079 (2)	0.192 (4)	−0.0311 (19)	0.061 (3)	0.003 (2)
O9	0.0541 (16)	0.0658 (17)	0.127 (2)	0.0163 (12)	0.0059 (15)	0.0145 (16)
C1	0.0335 (14)	0.0544 (18)	0.0443 (17)	0.0141 (13)	0.0033 (12)	0.0036 (13)
C2	0.0303 (14)	0.0530 (17)	0.0434 (16)	0.0157 (12)	0.0070 (12)	0.0056 (13)
C3	0.0363 (14)	0.0413 (16)	0.0421 (16)	0.0109 (12)	0.0004 (12)	0.0031 (12)
C4	0.0443 (16)	0.0460 (16)	0.0398 (16)	0.0161 (13)	0.0021 (13)	0.0025 (12)
C5	0.072 (2)	0.0535 (19)	0.0418 (18)	0.0111 (16)	0.0008 (16)	0.0014 (14)
C6	0.078 (3)	0.086 (3)	0.078 (3)	0.052 (2)	−0.011 (2)	−0.019 (2)
C7	0.0501 (17)	0.0454 (17)	0.0566 (19)	0.0240 (14)	0.0011 (14)	0.0076 (14)
C8	0.0538 (19)	0.062 (2)	0.059 (2)	0.0257 (16)	0.0027 (16)	0.0186 (16)
C9	0.067 (2)	0.070 (2)	0.0472 (19)	0.0258 (18)	0.0158 (16)	0.0065 (16)

### Geometric parameters (Å, º)

**Table:**

N1—C6	1.475 (4)	C1—H1B	0.9700
N1—C5	1.484 (4)	C2—H2A	0.9700
N1—C1	1.484 (3)	C2—H2B	0.9700
N1—H1	0.90 (3)	C3—C4	1.515 (4)
N2—C3	1.495 (3)	C3—H3A	0.9700
N2—C2	1.498 (3)	C3—H3B	0.9700
N2—C7	1.499 (3)	C4—H4A	0.9700
N2—H2	0.87 (3)	C4—H4B	0.9700
N3—C9	1.479 (4)	C5—H5A	0.9600
N3—C4	1.489 (4)	C5—H5B	0.9600
N3—C8	1.491 (4)	C5—H5C	0.9600
N3—H3	0.87 (3)	C6—H6A	0.9600
N4—O2	1.214 (4)	C6—H6B	0.9600
N4—O3	1.223 (4)	C6—H6C	0.9600
N4—O1	1.249 (4)	C7—H7A	0.9600
N5—O6	1.204 (3)	C7—H7B	0.9600
N5—O4	1.230 (3)	C7—H7C	0.9600
N5—O5	1.248 (3)	C8—H8A	0.9600
N6—O8	1.205 (4)	C8—H8B	0.9600
N6—O7	1.221 (4)	C8—H8C	0.9600
N6—O9	1.237 (4)	C9—H9A	0.9600
C1—C2	1.509 (4)	C9—H9B	0.9600
C1—H1A	0.9700	C9—H9C	0.9600
			
C6—N1—C5	111.1 (3)	C4—C3—H3A	109.3
C6—N1—C1	113.2 (3)	N2—C3—H3B	109.3
C5—N1—C1	109.8 (2)	C4—C3—H3B	109.3
C6—N1—H1	112 (2)	H3A—C3—H3B	107.9
C5—N1—H1	108 (2)	N3—C4—C3	110.5 (2)
C1—N1—H1	102 (2)	N3—C4—H4A	109.6
C3—N2—C2	111.8 (2)	C3—C4—H4A	109.6
C3—N2—C7	110.1 (2)	N3—C4—H4B	109.6
C2—N2—C7	112.4 (2)	C3—C4—H4B	109.6
C3—N2—H2	103.5 (18)	H4A—C4—H4B	108.1
C2—N2—H2	109.4 (18)	N1—C5—H5A	109.5
C7—N2—H2	109.2 (18)	N1—C5—H5B	109.5
C9—N3—C4	112.2 (2)	H5A—C5—H5B	109.5
C9—N3—C8	110.5 (2)	N1—C5—H5C	109.5
C4—N3—C8	110.9 (2)	H5A—C5—H5C	109.5
C9—N3—H3	104 (2)	H5B—C5—H5C	109.5
C4—N3—H3	109 (2)	N1—C6—H6A	109.5
C8—N3—H3	111 (2)	N1—C6—H6B	109.5
O2—N4—O3	121.6 (3)	H6A—C6—H6B	109.5
O2—N4—O1	120.7 (3)	N1—C6—H6C	109.5
O3—N4—O1	117.7 (3)	H6A—C6—H6C	109.5
O6—N5—O4	122.3 (3)	H6B—C6—H6C	109.5
O6—N5—O5	121.5 (3)	N2—C7—H7A	109.5
O4—N5—O5	116.2 (3)	N2—C7—H7B	109.5
O8—N6—O7	125.1 (4)	H7A—C7—H7B	109.5
O8—N6—O9	119.9 (3)	N2—C7—H7C	109.5
O7—N6—O9	114.9 (3)	H7A—C7—H7C	109.5
N1—C1—C2	111.4 (2)	H7B—C7—H7C	109.5
N1—C1—H1A	109.3	N3—C8—H8A	109.5
C2—C1—H1A	109.3	N3—C8—H8B	109.5
N1—C1—H1B	109.3	H8A—C8—H8B	109.5
C2—C1—H1B	109.3	N3—C8—H8C	109.5
H1A—C1—H1B	108.0	H8A—C8—H8C	109.5
N2—C2—C1	110.6 (2)	H8B—C8—H8C	109.5
N2—C2—H2A	109.5	N3—C9—H9A	109.5
C1—C2—H2A	109.5	N3—C9—H9B	109.5
N2—C2—H2B	109.5	H9A—C9—H9B	109.5
C1—C2—H2B	109.5	N3—C9—H9C	109.5
H2A—C2—H2B	108.1	H9A—C9—H9C	109.5
N2—C3—C4	111.7 (2)	H9B—C9—H9C	109.5
N2—C3—H3A	109.3		

### Hydrogen-bond geometry (Å, º)

**Table:**

*D*—H···*A*	*D*—H	H···*A*	*D*···*A*	*D*—H···*A*
N1—H1···O9^i^	0.90 (3)	2.04 (3)	2.868 (4)	153 (3)
N1—H1···O7^i^	0.90 (3)	2.14 (3)	2.937 (4)	147 (3)
N2—H2···O5	0.87 (3)	1.90 (3)	2.749 (3)	162 (3)
N2—H2···O4	0.87 (3)	2.42 (3)	3.134 (4)	139 (2)
N3—H3···O1^i^	0.87 (3)	2.00 (3)	2.789 (4)	149 (3)
N3—H3···O3^i^	0.87 (3)	2.28 (3)	3.073 (4)	152 (3)
C1—H1*A*···O7^ii^	0.97	2.38	3.293 (4)	156
C1—H1*B*···O4	0.97	2.45	3.232 (4)	137
C2—H2*B*···O5^iii^	0.97	2.52	3.386 (4)	149
C3—H3*B*···O1^ii^	0.97	2.44	3.308 (4)	148
C4—H4*A*···O5^iii^	0.97	2.40	3.301 (4)	155
C5—H5*C*···O8^iv^	0.96	2.40	3.305 (5)	156
C7—H7*A*···O6^v^	0.96	2.42	3.295 (4)	152
C8—H8*A*···O2^iv^	0.96	2.50	3.355 (5)	149
C9—H9*A*···O2^iv^	0.96	2.53	3.377 (5)	148

Symmetry codes: (i) *x*, *y*−1, *z*; (ii) *x*+1, *y*−1, *z*; (iii) *x*−1, *y*, *z*; (iv) *x*+1, *y*, *z*; (v) *x*−1, *y*−1, *z*.
